# Teaching the Communication of Diagnostic Uncertainty at Scale: Leveraging a Mobile App for Just-in-Time Instruction

**DOI:** 10.7759/cureus.88385

**Published:** 2025-07-20

**Authors:** Dimitrios Papanagnou, Abagayle Bierowski, Casey Morrone, Ridhima Ghei, Kristin L Rising, Danielle M McCarthy, Kyle T Formella, John A Vozenilek, Shruti Chandra, Nethra Ankam

**Affiliations:** 1 Emergency Medicine, Thomas Jefferson University, Philadelphia, USA; 2 Emergency Medicine, Northwestern University Feinberg School of Medicine, Chicago, USA; 3 Jump Simulation, OSF HealthCare, Peoria, USA; 4 Rehabilitation Medicine, Thomas Jefferson University, Philadelphia, USA

**Keywords:** communication training, diagnostic uncertainty, emergency medicine, medical simulation, undergraduate medical education

## Abstract

Diagnostic uncertainty is a reality of clinical care, particularly in emergency medicine. The ability to communicate this uncertainty to patients and families, however, remains underdeveloped in medical education curricula. Traditional simulation training is regarded as the gold standard for teaching difficult conversations, but resource constraints can limit access, especially for larger cohorts of learners. Faced with this challenge, we turned to a tool our team developed: the Uncertainty Communication mobile application (app). With over 200 medical students entering their fourth year of training, we used this app to deliver real-time, large-scale, skill-based learning, without simulation rooms, standardized patients, or small-group role-play. Students practiced communicating diagnostic uncertainty to patients, received immediate feedback, and reflected on patient-centered communication strategies. Their responses were generally positive. While not a formal study or intended to replace the role of traditional simulation training, our experience reaffirms how the intentional integration of mobile tools into medical training can introduce complex skills, like communicating diagnostic uncertainty, into scalable and practical learning solutions. As we train the next generation of physicians, such tools may offer educators some degree of flexibility when training large cohorts of students.

## Editorial

Diagnostic uncertainty is a daily reality in clinical practice, especially in emergency medicine, where physicians must make decisions with incomplete information. Yet, the skill of communicating diagnostic uncertainty to patients remains underdeveloped in most training programs [1]. While simulation modalities are often viewed as the gold standard for teaching challenging conversations [2], simulation-based education demands physical space, standardized patients, and/or skilled faculty facilitators, resources that are not always readily available, particularly when working with large student groups and class sizes. The impact of this availability is even greater on rural and remote medical training, as well as resource-limited educational settings. 

We encountered this challenge as we prepared to teach our rising fourth-year medical students about diagnostic uncertainty during the Gateway to Internship course, which prepares students for their transition into their final year of medical school. In years prior, we had delivered this session virtually over Zoom and made use of our ability to seamlessly use virtual breakout groups to support peer-to-peer role-plays with a student observer [3,4]. This year, however, with the decision to transition this session to in-person delivery, logistical constraints (e.g., lack of simulation rooms, limited faculty, and the transition to an in-person auditorium setting) necessitated a reimagined approach. We needed a scalable, interactive, and effective method for engaging over 200 students in reflection and skill-building without dividing them into smaller groups in order to meet the session learning objectives.

To meet this need, we adapted our workshop using the Uncertainty Communication mobile application (app), a free and open-access tool developed several years ago by our research team, to support real-time learning and feedback [5]. During the session, students learned about the prevalence of diagnostic uncertainty in the emergency department, particularly during patient discharge. Patients frequently leave with symptom-based diagnoses (e.g., “abdominal pain”) after dangerous conditions are ruled out, without a confirmed explanation for their symptoms. When the limits of diagnosis and next steps are not clearly communicated, patients may feel abandoned, confused, or fearful, an experience known to negatively impact their physical and/or psychological health.

After being prompted to download the free app on their respective personal devices during the in-person session, students worked through realistic clinical scenarios on the app independently, guided by a framework based on the Uncertainty Communication Checklist (UCC) [6,7]. Students were able to download either the Apple [8] or Android [9] versions of the Uncertainty Communication mobile app. The app introduces the user to a fictional physician robot (“DocBot”) who must undergo training to better communicate diagnostic uncertainty to its patients. Users are presented with five thematic chapters of fictional patient encounters (explaining actions, explaining tests and treatments, asking patients questions, giving patients instructions, and explaining that there is no answer to explain symptoms), each tied to the core communication domains from the UCC (e.g., explaining tests and treatments). The dialog for each encounter was written by our multidisciplinary team, comprised of emergency medicine physicians and medical educators. Patient encounters within the app are loosely based on real scenarios that are typical for cases of diagnostic uncertainty in the emergency department [5]. 

**Figure 1:**
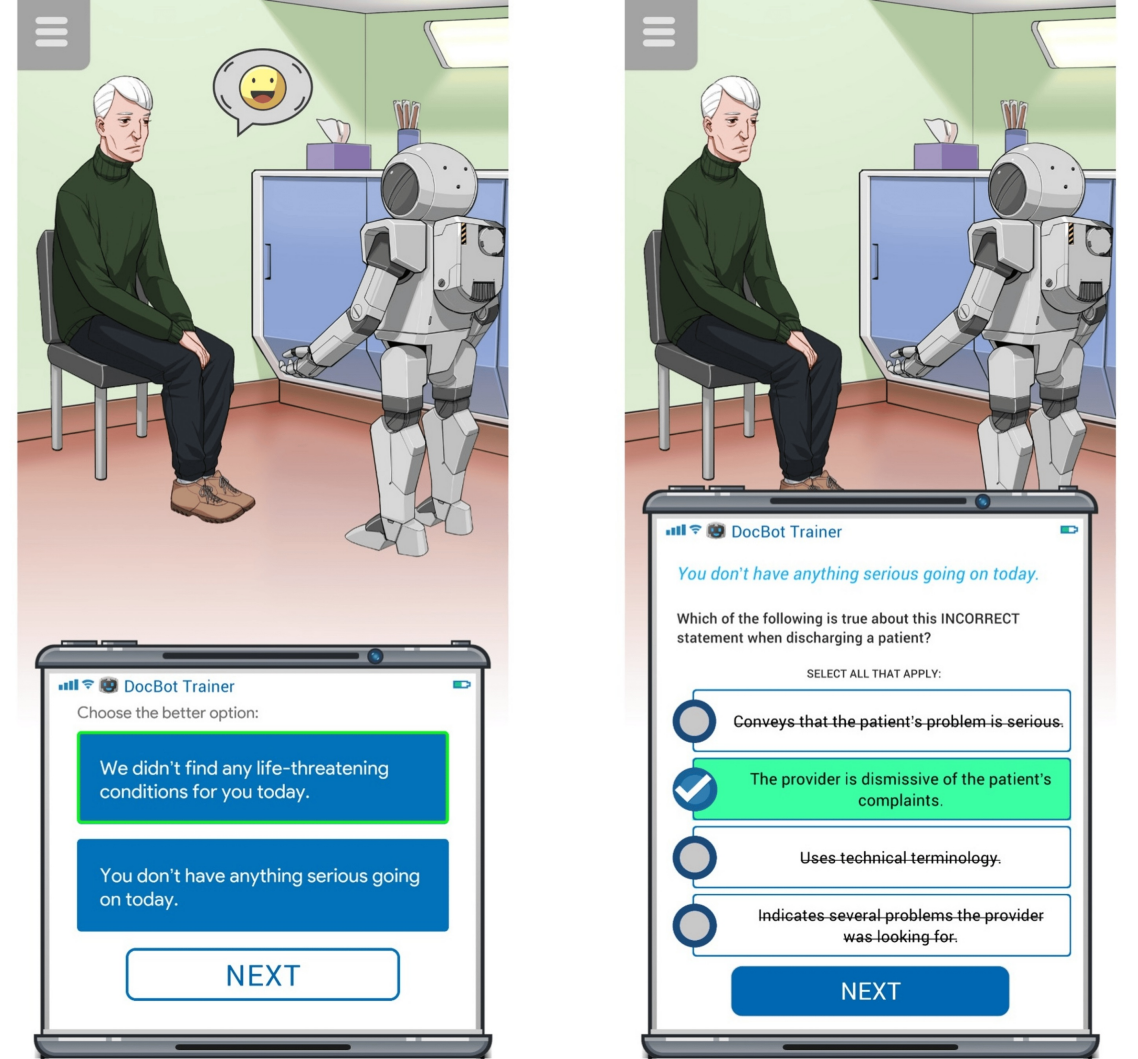
Example of fictional patient scenario in the Uncertainty Communication app

The app challenges users to make decisions on how to respond to specific patient questions during the emergency department discharge when there is no identified diagnosis to explain the patient's symptoms. Users of the app are prompted to select from several physician responses during the emergency department discharge and to identify the rationale for why a specific response was a more effective communication statement than the other presented options. The app experience is comprised of a repeating pattern of two questions: the first question (i.e., identifying the best communication statement) lists two options to select from, while the second question (i.e., the rationale for the best statement) lists four options to select from (Figure 1). Users are then provided with immediate formative feedback on their choices. Our overarching goal for incorporating this app into the session was to give students the space to reflect on language that reinforces transparency, trust, and patient-centered care during transitions in care (e.g., discharge from the emergency department). In doing so, we were able to deliver deliberate practice, feedback, and reflection at a large scale, without simulation rooms or in-person role-plays.

A key advantage of the app was its easy integration into a large-group lecture. It supported synchronous, just-in-time learning by allowing students to engage in meaningful reflection and immediate practice during the session itself. Feedback following the session was collected. The project was gauged to be exempt from Institutional Review Board (IRB) review. Of the >200 students in attendance, 53 completed the anonymous feedback survey. Responses were overwhelmingly positive: 85% of students found the app easy to use; 81% said the design made it easy to practice communication skills; 87% felt the app reinforced helpful phrases for navigating uncertainty; and 76% said they felt more prepared to communicate diagnostic uncertainty with patients.

Free-text comments praised the app’s concrete vignettes and examples, clarity around statements they should avoid, and usefulness in modeling realistic conversations. Students appreciated the “low-stakes,” self-paced nature of the exercise and the opportunity to learn specific and practical language. Suggestions for the app’s integration into the course included building in additional time for completing the app (as only 20 minutes was afforded for app engagement), as well as adding additional time to debrief the app as a large group for further reflection.

Although our work was not a formal study and did not collect any identifying student information, next steps will include conducting a formal investigation of the app's impact on students' ability to communicate diagnostic uncertainty. Our experience, however, illustrates how mobile tools can bridge the gap between theoretical instruction and practical application, particularly when physical, material, and/or operational logistics may constrain teaching. This may be helpful for other educators interested in similarly addressing uncertainty communication training for larger groups of learners. In preparing students for the uncertainty they will inevitably face in clinical practice, scalable tools like the Uncertainty Communication app offer a viable adjunct to our educational toolkit.
